# Myocardial iodine attenuation is associated with coronary artery stenosis severity: insights from photon-counting CT

**DOI:** 10.1007/s10554-026-03788-5

**Published:** 2026-08-12

**Authors:** M. C. Langenbach, K. Rippel, J. M. Brendel, I. L. Langenbach, H. Haltern, L. Schober, C. Scheurig-Muenkler, T. J. Kroencke, J. A. Decker

**Affiliations:** 1 Clinic of Diagnostic and Interventional Radiology, University Hospital Augsburg, Stenglin Str. 2 86156, Augsburg, Germany; 2 Cardiovascular Imaging Research Center (CIRC), Department of Radiology, Massachusetts General Hospital, Harvard Medical School, Boston, MA USA; 3 Department of Diagnostic and Interventional Radiology, University Hospital Tübingen, Tübingen, Germany

**Keywords:** Coronary Computed Tomography Angiography, CAD-RADS, Spectral Imaging, Myocardial Perfusion, Quantitative Imaging

## Abstract

To investigate whether myocardial iodine attenuation and iodine/virtual non-contrast (VNC) ratios derived from photon-counting detector CT (PCCT) are associated with coronary artery stenosis severity as classified by CAD-RADS, and to evaluate their potential complementary value to anatomical grading. In this retrospective single-center study, 57 patients (42.1% women; mean age 64.2 ± 13.0 years) who underwent clinically indicated coronary CT angiography using PCCT were included if they had either no coronary atherosclerosis or a measurable atherosclerotic lesion involving the left main or left anterior descending coronary artery, allowing iodine assessment in the corresponding LAD myocardial territory. Myocardial iodine attenuation was quantified on iodine maps using three circular regions of interest placed in the anterior left ventricular wall on mid-ventricular multiplanar reconstructions. Mean iodine attenuation and iodine/VNC ratios were calculated per patient. Coronary artery disease severity was graded according to CAD-RADS 2.0. Associations were assessed using Spearman correlation and linear regression analyses adjusted for age, body mass index, and VNC attenuation**.** Mean myocardial iodine attenuation was 32.1 ± 18.5 HU, and the mean iodine/VNC ratio was 0.69 ± 0.52. Both metrics showed an overall decrease with increasing CAD-RADS category, with the lowest values observed in patients with CAD-RADS 4 (iodine attenuation 16.4 ± 12.9 HU; iodine/VNC ratio 0.24 ± 0.15). Significant inverse correlations were observed between CAD-RADS category and iodine attenuation (r = -0.54, p < 0.001) as well as iodine/VNC ratio (r = -0.70, p < 0.001). In multivariable analyses, CAD-RADS category remained independently associated with iodine attenuation (β = -8.61 HU per category increase, p < 0.001) and iodine/VNC ratio (β = -0.14, p = 0.003). Myocardial iodine attenuation and iodine/VNC ratios derived from photon-counting CT are associated with coronary artery stenosis severity, classified by CAD-RADS, and may serve as quantitative adjuncts to anatomical coronary assessment. Further validation against established functional reference standards is required.

**Graphical abstract:**

**Figure:**
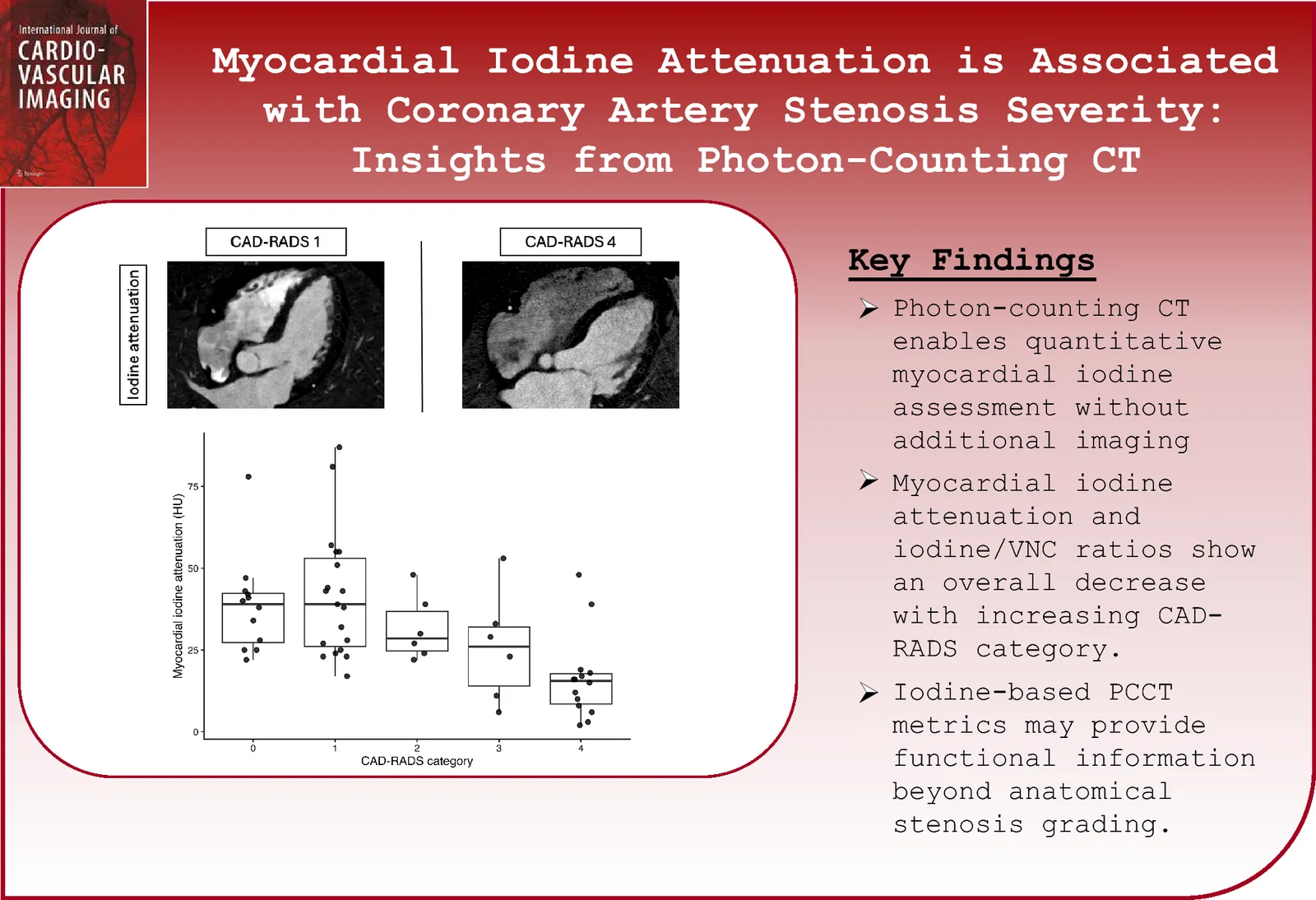

## Key points

• Photon-counting CT enables quantitative myocardial iodine assessment without additional imaging or scan time.
• Myocardial iodine attenuation and iodine/VNC ratios show an overall decrease with increasing CAD-RADS category.
• Iodine-based PCCT metrics may provide complementary quantitative information beyond anatomical stenosis grading but require validation against functional reference standards.

## Introduction

Coronary computed tomography angiography (CCTA) is a well-established, first-line noninvasive imaging test for the evaluation of suspected coronary artery disease (CAD) due to its excellent negative predictive value and ability to depict coronary anatomy with high spatial resolution [1, 2]. Despite these strengths, conventional energy-integrating detector CT (EID-CT) remains limited in the accurate assessment of stenosis severity, particularly in heavily calcified vessels, where blooming artifacts may artificially increase the apparent degree of luminal narrowing [3, 4]. As a consequence, the anatomical severity of stenosis often shows only a modest correlation with functional significance, with studies demonstrating that only approximately half of lesions graded as ≥ 50% stenosis on CCTA are hemodynamically significant on fractional flow reserve (FFR) [5, 6]. This diagnostic gap has led to the increasing clinical relevance of functional add-on techniques such as CT-derived FFR and CT perfusion, which are now incorporated into CAD-RADS 2.0 [3, 7]. However, these techniques require additional computational, imaging, or financial resources and are not universally available.

Photon-counting detector CT (PCCT) represents a major technological advancement with the potential to overcome several limitations of conventional CT. PCCT offers higher spatial resolution, reduced electronic noise, improved contrast-to-noise ratio, and inherently spectral imaging capabilities [8–10]. These advantages are particularly relevant in coronary imaging, where PCCT has demonstrated improved delineation of calcified plaques, reduced blooming, and enhanced visualization of the vessel lumen [11, 12]. Furthermore, PCCT enables precise quantification of iodine distribution via material-specific reconstruction, facilitating generation of iodine maps and virtual non-contrast (VNC) images [13].

Iodine attenuation within the myocardium or coronary lumen has emerged as a potentially informative quantitative imaging biomarker. Prior work with dual-energy CT has shown that myocardial iodine concentration correlates with perfusion and may help identify ischemia [14, 15]. Whether iodine signal measured downstream of a coronary stenosis on PCCT reflects lesion severity has not been fully investigated. Given the improved spectral fidelity of PCCT compared with dual-energy CT, iodine-based metrics may provide additional quantitative information related to downstream myocardial iodine distribution.

Thus, the objective of this study was to evaluate whether iodine attenuation derived from PCCT is associated with coronary stenosis severity according to CAD-RADS. We hypothesized that higher anatomical stenosis categories would be associated with reduced iodine attenuation in the corresponding myocardial territory.

### Materials and methods

#### Study design and population

This retrospective, single-center study was approved by the institutional review board with a waiver of informed consent due to minimal-risk use of existing clinical imaging data. Consecutive patients who underwent clinically indicated CCTA using a PCCT system between October 2024 and April 2025 were screened for eligibility. Patients were included if they had either no coronary atherosclerotic lesion (CAD-RADS 0) or a measurable atherosclerotic lesion involving the left main (LM) or left anterior descending (LAD) coronary artery, provided that image quality permitted quantitative iodine assessment in the corresponding LAD myocardial territory. Patients with atherosclerotic lesions confined to the right coronary artery or left circumflex coronary artery were excluded, because myocardial iodine attenuation was quantified in the anterior left ventricular wall corresponding to the LAD territory. Additionaly, patients with a total coronary occlusion (CAD-RADS 5) were not included in the evaluation. Exclusion criteria comprised prior myocardial infarction, previous coronary artery bypass grafting or stenting, and severe image artifacts (e.g., pacemaker leads) that interfered with myocardial iodine quantification.

Demographic and clinical data were extracted from electronic medical records. Coronary artery disease severity was graded according to CAD-RADS 2.0 by a level III and level I reader in consensus (MCL, ILL). For each patient, the highest CAD-RADS category in the left coronary territory was used to reflect maximal stenosis severity.

#### CT Acquisition protocol

All examinations were performed on a clinical PCCT scanner (NAEOTOM Alpha, Siemens Healthineers). Studies were acquired using prospectively ECG-triggered axial or retrospectively gated spiral modes depending on heart rate and clinical indication. Intravenous contrast medium with an iodine concentration of 350 mg/mL was administered at 6 mL/s, followed by a saline flush. Contrast volume was selected according to the institutional coronary CTA protocol (85–120 mL), and contrast timing was individualized using a test bolus strategy. Contrast timing was individualized using a test bolus strategy according to the institutional coronary CTA protocol.

Standard vendor-recommended cardiac photon-counting CT parameters were used, including a collimation of 0.4 mm, quantum iterative reconstruction, and energy threshold settings optimized for spectral analysis. Routine coronary CT angiography images, virtual non-contrast (VNC) images, and iodine maps were reconstructed using spectral material decomposition algorithms. For image analysis, virtual monoenergetic images at 70 keV reconstructed with a Qr40 kernel were used as the reference for attenuation measurements.

#### Image analysis

**Fig. 1:**
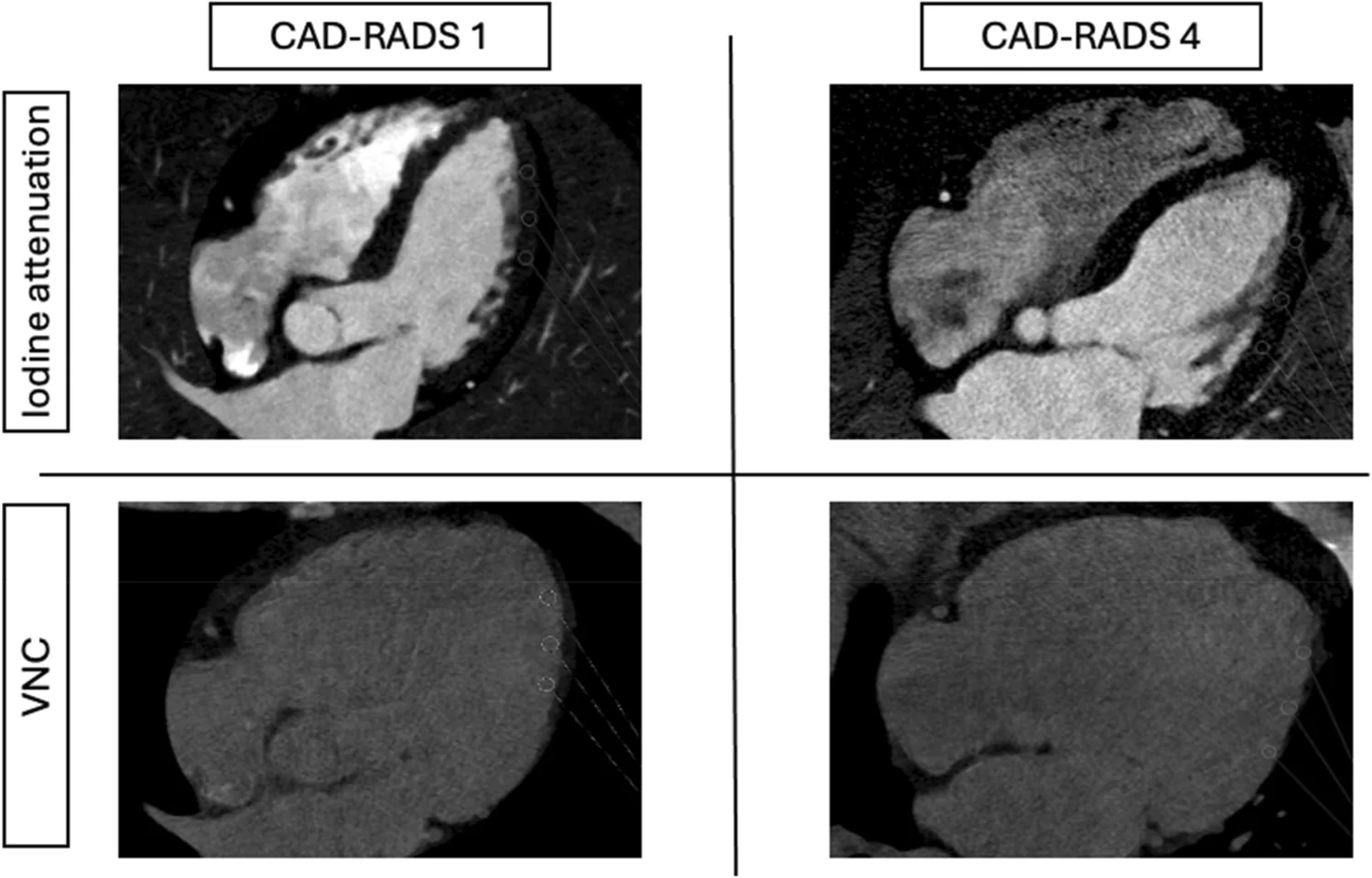
Representative photon-counting CT images in CAD-RADS 1 (left) and CAD-RADS 4 (right). Iodine maps (top) and corresponding VNC images (bottom) demonstrate reduced myocardial iodine attenuation in the LAD territory with increasing stenosis severity. CAD-RADS, Coronary Artery Disease-Reporting and Data System; VNC, virtual non-contrast

All image analyses were performed on a dedicated workstation (syngo.via, version 10.5, Siemens Healthineers). For iodine quantification, a trained reader blinded to CAD-RADS category placed three circular regions of interest (ROIs) within the anterior wall of the left ventricle, corresponding to the LAD myocardial territory. ROIs were positioned on mid-ventricular multiplanar reconstructions on iodine maps and carefully placed to avoid epicardial fat, papillary muscles, visible beam-hardening artifacts, and motion artifacts. Patients with severe artifacts interfering with myocardial iodine quantification were excluded from the analysis. Mean iodine attenuation (in Hounsfield units) across the three ROIs was calculated for each patient. The iodine/VNC ratio was additionally computed by dividing patient-level mean iodine attenuation by the corresponding attenuation measured on patient-specific VNC reconstructions. Measuring examples are displayed in Fig. 1.

#### Statistical analysis

Continuous variables were summarized as mean ± standard deviation or median with interquartile range, as appropriate. Categorical variables were reported as frequencies and percentages. Associations between CAD-RADS category (treated as an ordinal variable) and iodine parameters were assessed using Spearman’s rank correlation. Group-level comparisons of iodine attenuation and iodine/VNC ratio across CAD-RADS categories were performed using one-way ANOVA or Kruskal–Wallis testing as appropriate.

To evaluate whether CAD-RADS was independently associated with iodine metrics, multivariable linear regression models were constructed, adjusting for age, sex, body mass index (BMI), and myocardial VNC attenuation. VNC attenuation was included to account, in part, for interindividual differences in baseline myocardial attenuation. To assess the robustness of the findings against technical and physiological confounding, an extended sensitivity analysis additionally adjusted for heart rate, contrast volume, acquisition mode, and coronary dominance. Acquisition mode and coronary dominance were modeled as categorical variables. CAD-RADS was modeled as an ordinal variable, reflecting increasing stenosis severity per category. Regression coefficients (β) with 95% confidence intervals were reported. A two-sided p-value < 0.05 was considered statistically significant. All analyses were performed using R (R version 5.4.2, RStudio 2026.01.0 + 392) and SPSS (IBM, version 18.5).

## Results

A total of 131 patients who underwent cardiac CT with clinical suspicion of CAD were screened for inclusion. 13 patients were excluded due to a CAD-RADS 5 stenosis, 56 patients because of a coronary lesion other than the LM or LAD, and 5 patients due to non-evaluable stenosis degree due to image quality. Finally, a total of 57 patients, including 24 women (42.1%), were included in the analysis. The mean age was 64.2 ± 13.0 years, and the mean body mass index (BMI) was 27.2 ± 5.0 kg/m^2^. Mean myocardial iodine attenuation measured in the LAD territory was 32.1 ± 18.5 HU, while mean virtual non-contrast (VNC) attenuation was 53.0 ± 13.8 HU. The mean iodine/VNC ratio across the cohort was 0.69 ± 0.52 (Table 1).

**Table 1:** Baseline characteristics of the study population. BMI, body-mass index; CAD-RADS, coronary artery disease-reporting and data system; HU, Hounsfield units; VNC, virtual non-contrast

Characteristics	Value
n	57
Age, years	*64.2* ± *13.0*
Female, n (%)	*24 (42.1)*
Weight, kg	*81.9* ± *18.6*
Height, cm	*173.1* ± *10.0*
BMI, kg/m^2^	*27.2* ± *5.0*
CAD-RADS score distribution	0: n = 12, 1: n = 19, 2: n = 6, 3: n = 6, 4: n = 14
Iodine attenuation (HU)	32.1 ± 18.5
VNC attenuation (HU)	*53.0* ± *13.8*
Iodine/VNC ratio	0.69 ± 0.52

**Fig. 2:**
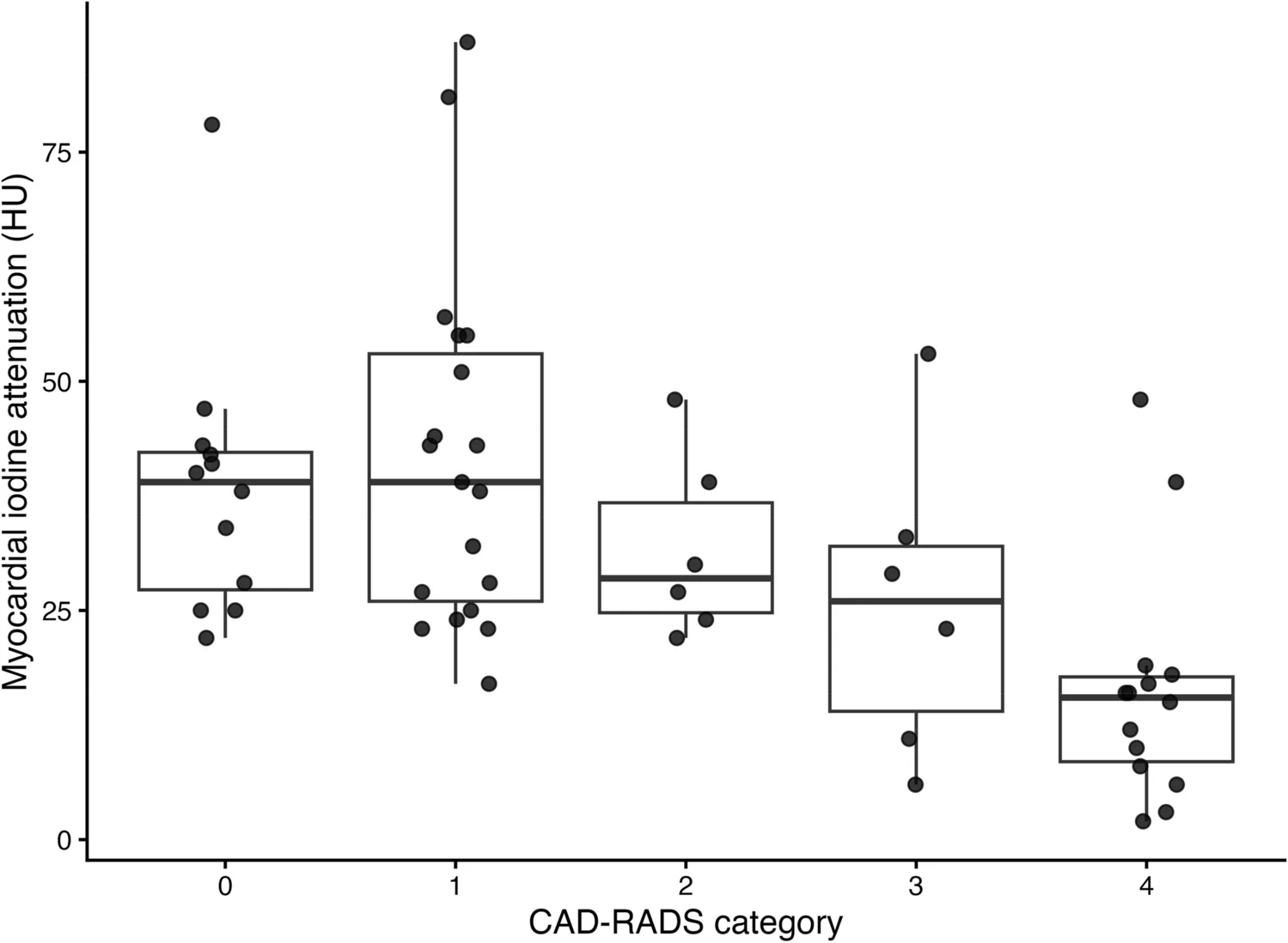
Distribution of myocardial iodine attenuation across CAD-RADS categories. Box-and-whisker plots with individual data points demonstrate overall decrease in myocardial iodine attenuation measured in the LAD territory with increasing CAD-RADS category. CAD-RADS, Coronary Artery Disease-Reporting and Data System; HU, Hounsfield units

When stratified by CAD-RADS category, myocardial iodine attenuation and iodine/VNC ratios showed an overall decrease with increasing stenosis severity. Group-level comparisons demonstrated significant differences across CAD-RADS categories for both myocardial iodine attenuation and the iodine/VNC ratio (Kruskal–Wallis p < 0.001 for both). The highest iodine attenuation values were observed in patients with CAD-RADS 0 and 1 (38.6 ± 14.9 HU and 41.7 ± 19.3 HU, respectively), with corresponding iodine/VNC ratios of 0.89 ± 0.47 and 1.00 ± 0.60. Lower values were observed in patients with CAD-RADS 2 and 3, with iodine attenuation values of 31.7 ± 10.0 HU and 25.8 ± 16.9 HU, and iodine/VNC ratios of 0.60 ± 0.16 and 0.40 ± 0.22, respectively. Patients with CAD-RADS 4 demonstrated the lowest iodine attenuation and iodine/VNC ratio (16.4 ± 12.9 HU and 0.24 ± 0.15, respectively). Given the modest sample size and small numbers in CAD-RADS categories 2 and 3, individual data points are displayed in Fig. 2 to illustrate the distribution of values within each category. Detailed values are provided in Table 2.

**Table 2:** Iodine Attenuation and Iodine/VNC Ratio by CAD-RADS Category. Values are presented as mean ± standard deviation. Overall group comparisons across CAD-RADS categories were performed using Kruskal–Wallis tests. CAD-RADS, Coronary Artery Disease-Reporting and Data System; HU, Hounsfield units; SD, standard deviation; VNC, virtual non-contrast

CAD-RADS Category	n	Iodine attenuation (HU), mean ± SD	Iodine/VNC ratio, mean ± SD
0	12	38.6 ± 14.9	0.89 ± 0.47
1	19	41.7 ± 19.3	1.00 ± 0.60
2	6	31.7 ± 10.0	0.60 ± 0.16
3	6	25.8 ± 16.9	0.40 ± 0.22
4	14	16.4 ± 12.9	0.24 ± 0.15
Overall p-value		< 0.001	< 0.001

**Fig. 3:**
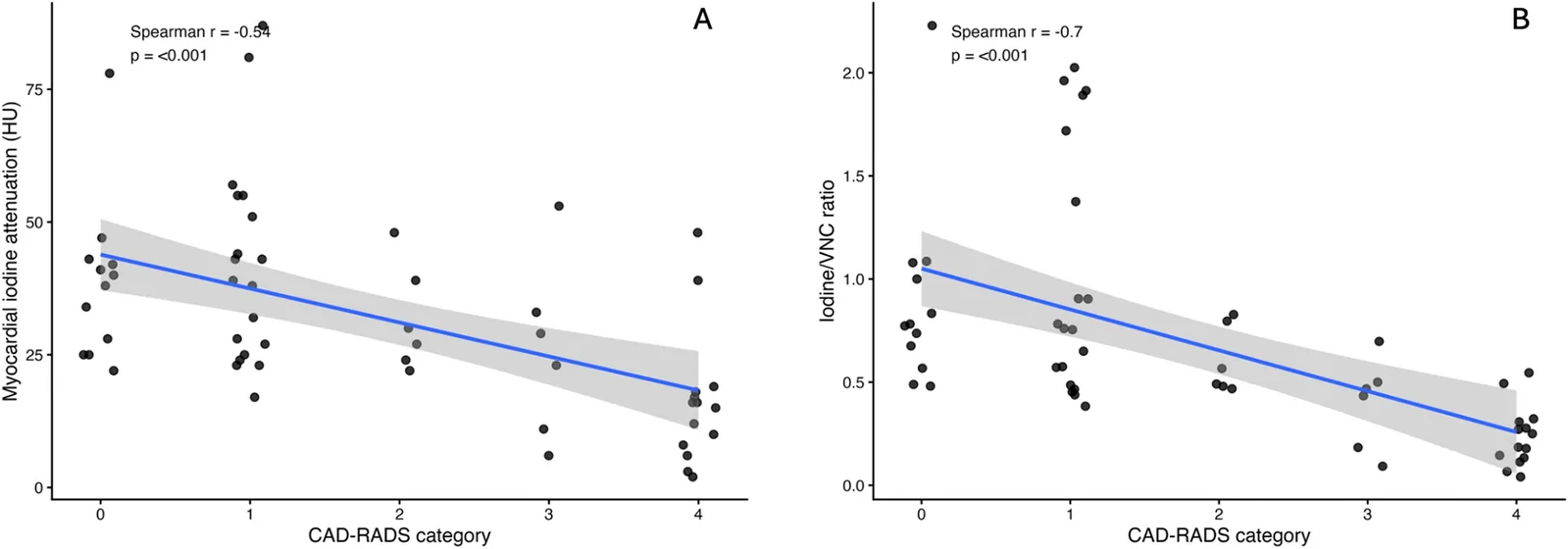
Association between CAD-RADS category and iodine-based myocardial metrics. Scatter plots with linear regression line (± 95% confidence band) demonstrates an inverse relationships between CAD-RADS category and iodine/VNC ratio (**A**) and myocardial iodine attenuation (**B**), consistent with reduced downstream iodine signal in higher stenosis categories. CAD-RADS, Coronary Artery Disease-Reporting and Data System; HU, Hounsfield units; VNC, virtual non-contrast

Spearman correlation analysis demonstrated a significant inverse relationship between CAD-RADS category and myocardial iodine attenuation (r = -0.54, p < 0.001) as well as between CAD-RADS and the iodine/VNC ratio (r = -0.70, p < 0.001). In univariable linear regression, each one-category increase in CAD-RADS was associated with a decrease of 6.40 HU in iodine attenuation (95% CI -9.22 to -3.57, p < 0.001) and a reduction of 0.20 in the iodine/VNC ratio (95% CI -0.27 to -0.12, p < 0.001). These associations remained significant after adjustment for age, sex, BMI, and VNC attenuation, with β = -8.61 HU (p < 0.001) for iodine attenuation and β = -0.14 (p = 0.003) for the iodine/VNC ratio (Table 3, Fig. 3). In an extended sensitivity analysis additionally adjusting for heart rate, contrast volume, acquisition mode, and coronary dominance, CAD-RADS category remained independently associated with iodine attenuation (β = -9.52 HU; 95% CI, -13.48 to -5.56; p < 0.001) and iodine/VNC ratio (β = -0.163; 95% CI, -0.268 to -0.059; p = 0.003).

**Table 3:** Univariable and multivariable regression analyses

Outcome variable	Model	β Coefficient	95% CI	p-value
Iodine attenuation (HU)	Univariable	-6.40	-9.22 to -3.57	< 0.001
	Multivariable	-8.61	-12.21 to -5.01	< 0.001
	Extended sensitivity model	-9.52	-13.48 to -5.56	< 0.001
Iodine/VNC ratio	Univariable	-0.20	-0.27 to -0.12	< 0.001
	Multivariable	-0.14	-0.24 to -0.05	0.003
	Extended sensitivity model	-0.16	-0.27 to -0.06	0.003

Primary multivariable models were adjusted for age, sex, body mass index, and VNC attenuation. Extended sensitivity models were additionally adjusted for heart rate, contrast volume, acquisition mode, and coronary dominance. Values are given per 1 CAD-RADS category increase. Multivariable models are adjusted for. *CI, confidence interval; HU, Hounsfield units; VNC, virtual non-contrast*

Overall, myocardial iodine attenuation and the iodine/VNC ratio derived from photon-counting CT demonstrated a strong and graded association with coronary artery stenosis severity as classified by CAD-RADS.

## Discussion

In this retrospective single-center study using photon-counting detector CT, myocardial iodine attenuation and the iodine/VNC ratio measured in the LAD territory demonstrated a strong and graded inverse association with coronary artery stenosis severity as classified by CAD-RADS. Patients with higher CAD-RADS categories exhibited lower downstream myocardial iodine signal, and these associations remained significant after adjustment for age, sex, body mass index, and VNC attenuation. These findings suggest that iodine-based quantitative metrics derived from PCCT may provide complementary information to anatomical stenosis grading. However, in the absence of an external functional reference standard, the functional significance of reduced myocardial iodine attenuation cannot be determined from the present data.

The observed decrease in myocardial iodine attenuation with increasing CAD-RADS category may reflect altered downstream iodine distribution in patients with higher anatomical stenosis severity. Importantly, this association remained significant in an extended sensitivity analysis adjusting for available technical and physiological factors, including heart rate, contrast volume, acquisition mode, and coronary dominance. However, because myocardial iodine attenuation can also be influenced by contrast timing, cardiac output, reconstruction parameters, beam-hardening, and motion, and because no external functional reference standard was available, this interpretation remains hypothesis-generating. Prior work using dual-energy CT has demonstrated that myocardial iodine concentration correlates with myocardial blood volume and perfusion, and can identify ischemic myocardial territories when compared with reference standards such as stress perfusion imaging and invasive FFR [14–17]. These studies established iodine as a quantitative surrogate of myocardial perfusion but were limited by spectral overlap and reduced signal-to-noise ratio, particularly in cardiac imaging.

Photon-counting CT overcomes several of these limitations by enabling direct photon energy discrimination, improved spectral separation, and more robust material decomposition [8–10, 18]. As a result, iodine quantification with PCCT is more precise and less susceptible to noise and beam-hardening artifacts than dual-energy CT. Our findings extend this prior work by demonstrating that iodine attenuation measured from routine coronary CT angiography, without stress imaging or additional acquisitions, correlates with coronary stenosis severity as graded by CAD-RADS.

Although coronary CT angiography provides excellent anatomical detail, the relationship between anatomical stenosis severity and functional significance is known to be modest, particularly for intermediate lesions and in the presence of heavy calcification [5, 6]. Advanced functional techniques such as CT-derived FFR and CT perfusion improve diagnostic accuracy and are now incorporated into CAD-RADS 2.0 [3, 7]. However, these approaches require additional computational resources, contrast administration, or radiation exposure, and are not universally available.

In contrast, iodine-based metrics derived from PCCT are obtained from the same standard CCTA acquisition without additional scan time or patient burden. The strong independent association between CAD-RADS category and iodine attenuation observed in our multivariable analyses suggests that PCCT-derived iodine metrics may help refine lesion characterization, particularly in patients with anatomically intermediate stenoses. Notably, the iodine/VNC ratio demonstrated an even stronger correlation with CAD-RADS than absolute iodine attenuation, potentially reflecting normalization for baseline myocardial attenuation and interindividual variability.

From a clinical perspective, myocardial iodine quantification with PCCT may represent a practical quantitative adjunct to anatomical coronary assessment. If validated in future studies against functional reference standards, quantitative iodine mapping may help identify patients in whom additional functional testing or invasive evaluation could be considered. As PCCT systems become increasingly available in clinical practice, incorporation of iodine-based myocardial assessment into routine CCTA workflows may enhance quantitative lesion characterization without increasing scan complexity.

This study has several limitations. First, its retrospective single-center design and modest sample size may limit generalizability. Second, iodine attenuation was assessed only in the LAD territory, and findings may not be directly applicable to other coronary distributions. Third, no invasive FFR, stress perfusion CT, SPECT/PET, cardiac MRI perfusion, or clinical outcome data were available for direct validation of functional significance. Therefore, the present study cannot determine whether reduced myocardial iodine attenuation reflects ischemia, impaired perfusion, or lesion-specific hemodynamic relevance. The findings should be interpreted as an association between anatomical CAD-RADS severity and iodine-based myocardial metrics. Fourth, CAD-RADS was modeled as an ordinal variable; although this reflects increasing stenosis severity, categorical modeling may provide additional granularity in larger cohorts. Finally, variability in iodine/VNC ratios was partly driven by low VNC attenuation values in a small number of patients.

Future multicenter studies should validate these findings in larger populations and directly compare PCCT-derived iodine metrics with established functional reference standards. Longitudinal studies evaluating the prognostic value of myocardial iodine attenuation for adverse cardiovascular outcomes are also warranted. Advances in automated or AI-based myocardial iodine quantification may further facilitate clinical adoption.

## Conclusion

Myocardial iodine attenuation and the iodine/VNC ratio derived from photon-counting CT demonstrate a strong and independent association with coronary artery stenosis severity as classified by CAD-RADS. These findings support the potential role of iodine-based PCCT metrics as noninvasive quantitative adjuncts to anatomical coronary assessment and highlight the added value of spectral imaging in contemporary cardiac CT. Further validation against functional reference standards is required before these metrics can be interpreted as markers of ischemia or hemodynamic significance.

## Data Availability

No datasets were generated or analysed during the current study.
